# Evaluating Care Innovations: A RCT of Home Teleassistance for Older Adults in Need of Care in Costa Rica

**DOI:** 10.1093/geroni/igaf122.2688

**Published:** 2025-12-31

**Authors:** Alexander Chaverri Carvajal

**Affiliations:** Universitat Autònoma de Barcelona, Bellaterra, Barcelona, Spain

## Abstract

Costa Rica faces increasing pressure to develop Long-Term Care services for dependent individuals. In 2022, the government enacted a law granting care rights to dependent older adults. However, a comprehensive nationwide network of residential, home-based, or teleassistance services has yet to be implemented. In 2024, the local government of Heredia initiated a pilot of home teleassistance program for dependent older adults, assessing their situation before and after service delivery. This study aimed to evaluate the impact of the home teleassistance pilot on four key areas: (a)improving quality of life for dependent individuals, (b)reducing unnecessary hospital visits, (c)promoting correct medication use, and (d)caregiver outcomes, including burnout and workforce participation among family caregivers. A randomized controlled trial (RCT) was conducted with 634 dependent older adults and their family caregivers. Participants were randomly assigned to receive home teleassistance services or remain in a control group. A structured questionnaire assessed the four dimensions outlined in the objectives. Home teleassistance improved quality of life of care recipients, reduced caregiver burnout, improved medication use and decreased hospital visits. Additionally, the program showed limited impact in enabling labor participation among caregivers. The findings underscore the potential of teleassistance programs to enhance Costa Rica’s long-term care (LTC) system. However, it is important to note that LTC systems typically integrate such programs into a broader care framework that includes home-based and residential care services. These insights offer critical guidance for the design and expansion of LTC strategies in Costa Rica and similar aging societies.

